# Retraction Note: 11,670 whole-genome sequences representative of the Han Chinese population from the CONVERGE project

**DOI:** 10.1038/s41597-020-0430-x

**Published:** 2020-04-16

**Authors:** Na Cai, Tim B. Bigdeli, Warren W. Kretzschmar, Yihan Li, Jieqin Liang, Jingchu Hu, Roseann E. Peterson, Silviu Bacanu, Bradley Todd Webb, Brien Riley, Qibin Li, Jonathan Marchini, Richard Mott, Kenneth S. Kendler, Jonathan Flint

**Affiliations:** 1 Wellcome Trust Centre for Human Genetics, OX3 7BN Oxford, UK; 2 Wellcome Trust Sanger Institute, Wellcome Genome Campus, CB10 1SA Hinxton, Cambridge UK; 3 European Molecular Biology Laboratory, European Bioinformatics Institute, Wellcome Genome Campus, CB10 1SD Hinxton, Cambridge UK; 4 Virginia Institute for Psychiatric and Behavioral Genetics, Virginia Commonwealth University, Richmond, Virginia 23298 USA; 5 BGI-Shenzhen, Shenzhen, Guangdong, 518083 China; 6 Department of Statistics, University of Oxford, Oxford, OX1 3TG UK; 7 UCL Genetics Institute, University College London, London, WC1E 6BT UK; 8 Center for Neurobehavioral Genetics, Semel Institute for Neuroscience and Human Behavior, University of California, LosAngeles, Los Angeles, California 90095-1761 USA

Retraction of: *Scientific Data* 10.1038/sdata.2017.11, published online 14 February 2017

The authors are retracting this Data Descriptor because the whole genome sequencing data associated with this study are no longer available through public repositories. As these data are no longer available to other researchers, we feel that the purpose of this Data Descriptor is sufficiently undermined to require retraction. These data were withdrawn from the INSDC repositories after publication at the request of the sequencing centre, BGI, after a change in data sharing regulations in China. At this time, genotype data and other processed data files remain available through the European Variation Archive and figshare. All authors agree to this Retraction.

